# Supplementary material for: Development of High-Resolution Dedicated PET-Based Radiomics Machine Learning Model to Predict Axillary Lymph Node Status in Early-Stage Breast Cancer
Source: Cancers (Basel). 2022 Feb 14;14(4):950. doi: 10.3390/cancers14040950 (PMC8870230; doi:10.3390/cancers14040950)
Supplement: Supplementary file 1 [file cancers-14-00950-s001.zip › cancers-1528297-supplementary.pdf]

Table S1. Specific categories of radiomics features.

Matrix	Radiomics Feature Name	Abbreviations
Shape features (n = 14)	Elongation	/
	Flatness	/
	Least Axis	/
	Major Axis	/
	Maximum 2D Diameter Column	M2DDC
	Maximum 2D Diameter Row	M2DDR
	Maximum2D Diameter Slice	M2DDS
	Maximum3D Diameter	M3DD
	Mesh Volume	/
	Minor Axis	/
	Sphericity	/
	Surface Area	/
	Surface Volume Ratio	SVR
	Volume	/
First-order features (n = 18)	10 Percentile	/
	90 Percentile	/
	Energy	/
	Entropy	/
	Interquartile Range	/
	Kurtosis	/
	Maximum	/
	Mean Absolute Deviation	MAD
	Mean	/
	Median	/
	Minimum	/
	Range	/
	Robust Mean Absolute Deviation	RMAD
	Root Mean Squared	RMS
	Skewness	/
	Total Energy	/
	Uniformity	/
	Variance	/
Gray Level Co-occurrence Matrix (GLCM) (n = 24)	Autocorrelation	/
	Cluster Prominence	CP
	Cluster Shade	CS
	Cluster Tendency	CT
	Contrast	/
	Correlation	/
	Difference Average	DA
	Difference Entropy	DE
	Difference Variance	DV
	Id	/
	Idm	/
	Idmn	/
	Idn	/
	Imc1	/
	Imc2	/

	Inverse Variance	/
	Joint Average	/
	Joint Energy	/
	Joint Entropy	/
	MCC	/
	Maximum Probability	/
	Sum Average	/
	Sum Entropy	/
	Sum Squares	/
Gray Level Dependence Matrix (GLDM) (n = 14)	Dependence Entropy	DE
	Dependence Non-Uniformity	DNU
	Dependence Non-Uniformity Normalized	DNUN
	Dependence Variance	DV
	Gray Level Non-Uniformity	GLNU
	Gray Level Variance	GLV
	High Gray Level Emphasis	HGLE
	Large Dependence Emphasis	LDE
	Large Dependence High Gray Level Emphasis	LDHGLE
	Large Dependence Low Gray Level Emphasis	LDLGLE
	Low Gray Level Emphasis	LGLE
	Small Dependence Emphasis	SDE
	Small Dependence High Gray Level Emphasis	SDHGLE
	Small Dependence Low Gray Level Emphasis	SDLGLE
Gray Level Run Length Matrix (GLRLM) (n = 16)	Gray Level Non-Uniformity	GLNU
	Gray Level Non-Uniformity Normalized	GLNUN
	Gray Level Variance	GLV
	High Gray Level Run Emphasis	HGLRE
	Long Run Emphasis	LRE
	Long Run High Gray Level Emphasis	LRHGLE
	Long Run Low Gray Level Emphasis	LRLGLE
	Low Gray Level Run Emphasis	LGLRE
	Run Entropy	RE
	Run Length Non-Uniformity	RLNU
	Run Length Non-Uniformity Normalized	RLNUN
	Run Percentage	RP
	Run Variance	RV
	Short Run Emphasis	SRE
	Short Run High Gray Level Emphasis	SRHGLE
	Short Run Low Gray Level Emphasis	SRLGLE
Gray Level Size Zone Matrix (GLSZM) (n = 16)	Gray Level Non-Uniformity	GLNU
	Gray Level Non-Uniformity Normalized	GLNUN
	Gray Level Variance	GLV
	High Gray Level Zone Emphasis	HGLZE
	Large Area Emphasis	LAE
	Large Area High Gray Level Emphasis	LAHGLE
	Large Area Low Gray Level Emphasis	LALGLE
	Low Gray Level Zone Emphasis	LGLZE
	Size Zone Non-Uniformity	SZNU
	Size Zone Non-Uniformity Normalized	SZNUN
	Small Area Emphasis	SAE

	Small Area High Gray Level Emphasis	SAHGLE
	Small Area Low Gray Level Emphasis	SALGLE
	Zone Entropy	ZE
	Zone Percentage	ZP
	Zone Variance	ZV
Neighboring Gray Tone Difference Matrix (NGTDM) (n = 5)	Busyness	/
	Coarseness	/
	Complexity	/
	Contrast	/
	Strength	/
Wavelet features (n = 744)	LLH (n = 93)	WLLH
	LHL (n = 93)	WLHL
	LHH (n = 93)	WLHH
	HLL (n = 93)	WHLL
	HLH (n = 93)	WHLH
	HHL (n = 93)	WHHL
	HHH (n = 93)	WHHH
	LLL (n = 93)	WLLL
